# *Tenebrio molitor* (Linnaeus, 1758): Microbiological Screening of Feed for a Safe Food Choice

**DOI:** 10.3390/foods12112139

**Published:** 2023-05-25

**Authors:** Barbara Pöllinger-Zierler, Andrea Lienhard, Chiara Mayer, Simon Berner, René Rehorska, Angela Schöpfer, Monika Grasser

**Affiliations:** Sustainable Food Management, Institute of Applied Production Sciences, Department of Engineering, University of Applied Sciences, FH JOANNEUM GmbH, Eggenberger Allee 11, 8020 Graz, Austria; barbara.poellinger-zierler@fh-joanneum.at (B.P.-Z.);

**Keywords:** larvae of *Tenebrio molitor*, yellow mealworm, by-products from food production, microbial load, alternative protein source, safe food choice, sustainability, reuse of by-products

## Abstract

As a result of the increasing focus on alternative protein sources which are ideally still sustainable, the yellow mealworm, *Tenebrio molitor*, has come into focus. To verify its suitability as a food source in relation to human health, an analysis of the microbiome of larvae of *T. molitor* is pertinent. Subsequently, the focus of this study was, on the one hand, to analyze the influence of the substrate on the microbial load of the larvae microbiome, and, on the other hand, to determine which processing methods ensure the risk-free consumption of mealworms. For this purpose, mealworms were grown on 10 different substrates derived from by-products of food production (malt residual pellets, corn germ meal, chestnut breakage and meal, wheat bran, bread remains, draff, nettle, hemp seed oil cake, oyster mushrooms with coffee grounds, pumpkin seed oil cake) and microbial loads were analyzed using different selective media. Further starvation/defecation and heating (850 W for 10 min) methods were used to investigate how the reduction of microorganisms is enabled by these methods. The results showed that there was no significant relationship between the microbial load of the substrate and the mealworm. Starvation and defecation led to a lower stock of microorganisms. Heating led to a significant microbial reduction in non-defecated mealworms. The group of defecated and heated mealworms showed no detectable microbial load. In conclusion, firstly, the choice of substrate showed no effect on the microbial load of larvae of *Tenebrio molitor* and secondly, heating and starvation allow risk-free consumption. This study makes an important contribution for evaluating the safety of mealworms as a sustainable protein source in human nutrition.

## 1. Introduction

As the demand for food continues to grow, so has the desire and search for alternative food sources. In order to eat sustainably, behaviors such as a preference for plant-based food, organically produced food, regional and seasonally produced food, or adopting a resource-conserving form of housekeeping are recommended. In addition to these recommendations, however, the food industry is also on the lookout for sustainable, new sources that will provide essential protein to the ever-growing population on the planet. In this context, edible insects are gaining more and more attention as a sustainable food source which is not plant-based and serves as a high-quality source of protein.

In general, insects use less land than conventional livestock, reproduce much faster, have higher feed conversion efficiencies, and produce lower greenhouse gas emissions [1]. Moreover, insects are highly nutritious and represent a good source of protein (with a well-balanced amino acid profile), fat and micronutrients like vitamins and minerals. However, insect consumption, especially in Western countries, is still limited, not only because of the unpleasant perception that many people have of insects but also because of regulations regarding safety and hygiene issues [2]. As is the case for conventionally produced food products, it is important to monitor possible safety hazards for insect-derived foods as well [3]. Potential food safety hazards concerning edible insects are toxins (especially mycotoxin and bacterial toxins), heavy metals, chemical hazards (pesticides), and allergens [4,5]. Most of the insect microbiota are associated with the gut, but there is also a risk from extrinsic sources, such as the environment, and rearing, handling, processing, and preservation conditions [5,6].

Larvae of the yellow mealworm (*Tenebrio molitor* Linnaeus, 1758), which belong to the family of darkling beetles (Tenebrionidae), are favorable candidates for insect rearing and have thus gained increased attention as a protein source. Mealworms were categorized as “Novel Foods” (Novel Food Regulation (EU) No 2015/2283) and in June 2021, the EFSA submitted the first completed evaluation of insect-derived food made from mealworm larvae. Despite this evaluation, more information on their safety is imperative. The potential safety risks of eating mealworms need to be identified for legislators, markets, and consumers [6,7]. One of the potential risks associated with the consumption of this insect species is the possible presence of microbial food pathogens, because there is still a lack of profound insight into the microbiological safety of such consumption in general, for example, studies on the microbiological load of larvae [2,3]. Studies focusing on the microbial contamination in fresh *Tenebrio molitor* samples have been conducted by several authors [7,8,9,10,11,12,13], but only a few research studies investigated the effect of different feeding substrates and rearing conditions on the chemical–biological characteristics of mealworm larvae. Nevertheless, the plasticity of *T. molitor* in relation to different substrates with variations of microbiological loads has been highlighted by Mancini et al. [13]. Generally, the microbial load of fresh mealworms was found to be high, with total viable counts of about 5.5–8.5 log cfu × g^−1^ [8,9,10,12,13,14,15]. Therefore, a processing step to reduce the number of microorganisms is recommended by the scientific community.

According to Wynants et al., many studies do not provide information on the used substrate, which most often consists of wheat bran supplemented with carrots or other moisture-rich components [3], although it has been suggested that the substrate, along with the rearing techniques, the environment, and the hygiene affect the (gut) microbiota of *T. molitor* [3,8,16].

The aim of this study was to investigate the microbiological load of mealworm larvae (*Tenebrio molitor*) in different conditions (starved and unstarved and therefore defecated and non-defecated, heated and non-heated) and grown on ten different rearing substrates. Larvae were fed with several local by-products (chestnut breakage and meal, malt residual pellets, wheat bran, bread, draff, nettle, hemp, oyster mushrooms with coffee grounds, corn germ meal, and pumpkin seed oil cake) and the effects of the microbiological load were examined. The focus was placed firstly on whether the reuse of by-products from food production is suitable as a breeding basis and, secondly, whether the microbial load of mealworms enables their use as a food source.

## 2. Materials and Methods

### 2.1. Insect Rearing Conditions

Mealworm last instar larvae were obtained from the *Tenebrio molitor* colony, which had been reared since 2019 at the University of Applied Sciences (FH JOANNEUMGmbH, Graz, Austria). The insects were reared in plastic boxes under optimal conditions (~27 °C, 65% RH) in a growth chamber (KBF-S, WTC Binder GmbH, Tuttlingen, Germany).

To investigate the influence of different growing media on the mealworm microbiome, they were fed on different substrates for several generations. Ten by-product streams were selected for *T. molitor* larvae based on the following criteria described by Derler et al. [17]: resource- and cost-efficiency in mass rearing, being free from any harmful contaminants, locally available in large quantities, logistically easy to handle, and storable with a long shelf-life. One growing box was used for each substrate. The cultivation boxes with the different substrates were all stored in the cultivation cabinet under the same rearing conditions. The following substrates were applied: malt residual pellets (MRP), corn germ meal (CGM), chestnut breakage and meal (CM), wheat bran (WB), bread remains (B), draff (D), nettle (N), hemp seed oil cake (H), oyster mushrooms with coffee grounds (OMCG), and pumpkin seed oil cake (PSOC). The following substrates were dried using a drying cabinet (TS540WTC Binder GmbH Tuttlingen, Germany)) between 50 and 70 °C before use: nettle, bread remains, draff, oyster mushrooms with coffee grounds, and chestnut breakage. Nettle stalks were removed, and the leaves were manually crushed before feeding. Bread remains, hemp seed oil cake, oyster mushrooms with coffee grounds, malt residual pellets, and pumpkin seed oil cake were ground by a blender before feeding (VM0105E, Vitamix, Frankfurt, Germany).

The water activity was determined using ISO 18787 (LabMaster Neo, Novasina, Lachen, Switzerland) for all substrates except draff due to lack of quantity. To determine the pH value (pH720/SenTix 41, WTW GmbH, Weilheim in Upper Bavaria, Germany)), 5 g of each substrate (except draff, due to missing quantity) was mixed with 45 g of deionized water for 10 min. Due to the large volume of nettle, double the amount of water was used.

As a result of the experiments concerning the influence of different substrates on the microbial load of the larvae of *T. molitor*, different processing methods were utilized to reduce this microbial load and thus generate mealworms grown on these substrates as a safe food. The influence of starvation, feed withdrawal, and heating were investigated as processing steps to reduce the presence of microorganisms on the larvae of yellow mealworm. For this experiment, larvae were cultivated on wheat bran. Subsequently, they were divided into four groups to be analyzed: starvation and defecation for 24 h and non-heated (D/NH), starvation and defecation for 24 h and heated (D/H), non-defecated and heated (ND/H), and non-defecated and non-heated (ND/NH). To evaluate a potential microbial reduction, heating of the mealworm larvae was performed using a microwave (850 W for 10 min (NE-1027, Panasonic, Wiesbaden, Germany), according to Böschen et al. [18]. All samples were collected using sterile plastic bags with sterile instruments.

### 2.2. Bacterial Counts

For the microbial analysis, 2.5 g of (i) different substrates, (ii) fresh mealworm larvae (corresponding to approximately 60 insects) taken directly from the substrate, and (iii) every larvae batch from the starvation and heating experiment were used. The mealworm larvae were cut into pieces using an aseptic scalpel. Substrates and mealworm larvae were suspended in 20 mL of sterile peptone water (buffered peptone water, EN ISO 6887, Carl Roth, Karlsruhe, Germany) and homogenized twice for 30 s using a test tube shaker (Reax top Heidolph, Schwabach, Germany,). The resulting suspensions were diluted 10-fold and 100 µL per sample was subjected to microbial enumeration of total mesophilic aerobic bacterial counts (TAC), Enterobacteriaceae (EB), and yeasts and molds (YM) in the appropriate growth media. 3M™ Petrifilm™ (3M Science, Neuss, Germany) was used for the enumeration of Enterobaceriaceae and SGC2 agar plates (Sabouraud Gentamicin Chloramphenicol 2 Agar by Biomerieux, Vienna, Austria) to detect yeasts and molds. For the evaluation of the total mesophilic aerobic bacterial counts, COS agar plates (Columbia Serum Agar with 5% sheep blood by Biomerieux, Vienna, Austria) were used. Microbiological analyses were performed four times. The results of the microbial counts were expressed as the mean of log colony-forming units (cfu) per gram of sample ± standard deviation.

### 2.3. Statistical Analysis

The values obtained from the analyses were expressed as mean ± standard deviation. The data were analyzed using one-way analysis of variance (ANOVA) and Tukey’s post hoc test with a significance level of 0.05.

## 3. Results

This study was conducted to evaluate the microbial load of mealworms to generate a safety assessment regarding their suitability as a safe, sustainable protein food source.

- Influence of substrates on the microbial load of mealworm larvae

Ten different substrates were chosen, which play a role mainly as side streams or by-products in food production and represent a significant, sustainable alternative when used as rearing substrates. In Table 1, the microbial load (in log cfu × g^−1^) of all substrates and also of larvae of *Tenebrio molitor* grown on these substrates is shown. With repeated analysis, representative sampling from the breeding boxes, and the use of a high number of mealworms, the microbial load of larvae of the yellow mealworm could be evaluated. Enterobacteriaceae were found in all substrates; yeasts and molds were only detected in bread, hemp, and wheat bran substrates in a very low amount. Only corn germ meal and wheat bran showed a higher microbial load. The higher concentration of microorganisms could be explained by better and faster availability of the ingredients for microbial metabolism.

**Figure 1:**
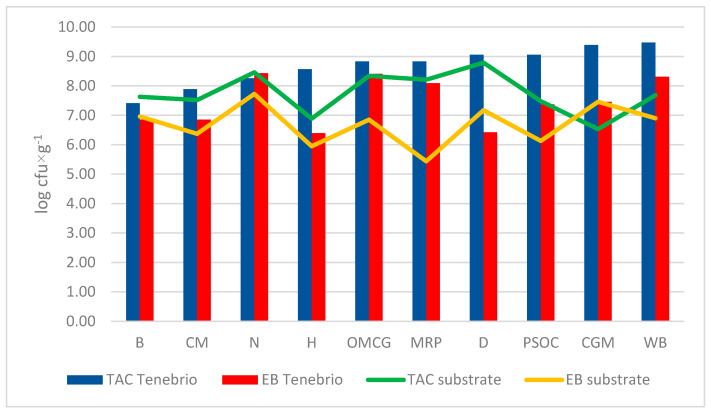
Analysis of a potential correlation between the number of viable and active microorganisms in substrates and larvae of *Tenebrio molitor* grown on these substrates. Abbr.: (N) nettle, (B) bread remains, (D) draff, (H) hemp seed oil cake, (OMCG) oyster mushrooms with coffee grounds, (WB) wheat bran, (CM) chestnuts breakage and meal, (CGM) corn germ meal, (MRP) malt residual pellets, (PSOC) pumpkin seed oil cake, (TAC) total mesophilic aerobic bacterial counts, (EB) Enterobacteriaceae.

According to the results of ANOVA (F 10.57; p 3.87 × 10^−16^) there was no statistically reliable correlation between the concentration of microorganisms on the substrate and that in the mealworm larvae. In addition, Figure 1 underlines the non-existent linear correlation.

- Influence of different processing methods on the microbial load of mealworm larvae

**Figure 2:**
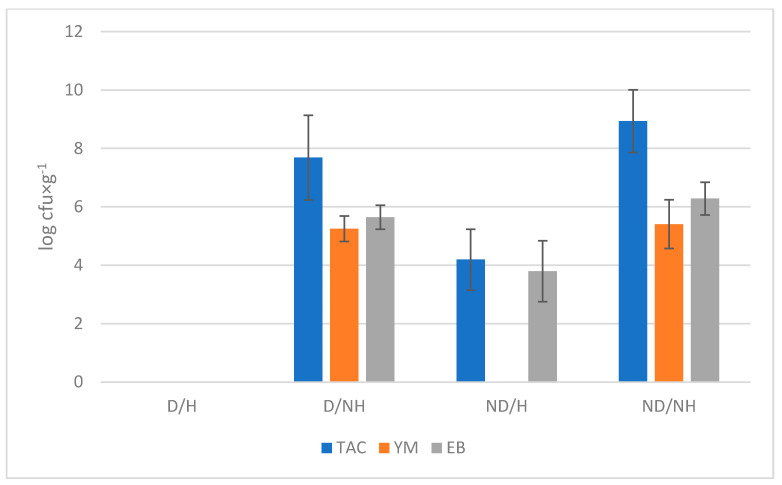
Microbial analysis of the of larvae of *Tenebrio molitor* which were defecated, non-defecated, heated, and non-heated. Abbr.: (D) defecated, (H) heated, (ND) non-defecated, (NH) non-heated, (TAC) total mesophilic aerobic bacterial counts, (YM) yeasts and molds, (EB) Enterobacteriaceae.

In order to consider mealworm larvae from a microbiological point of view as a safe food source, different processing steps were evaluated to analyze the potential reduction of microorganisms. Heating (850 W for 10 min) and starvation according to defecation (for 24 h) were designated as simple and reliable methods in this context. Table 2 and Figure 2 show that both processing methods led to a significant reduction in microorganisms, both performed individually and in combination. Starvation resulted in a significant reduction in microbial load, while heating the mealworms resulted in an even better reduction in microbial concentrations. If mealworms were not starved but heated, low concentrations of microorganisms were detectable. When the thermosensitive yeasts and molds were no longer visible, however, the more resistant Enterobacteriaceae as well as the total aerobic mesophilic microorganisms showed values of 4.19 ± 1.04 log cfu × g^−1^. Non-starved and non-heated mealworm larvae had the highest concentrations of microorganisms including aerobic mesophilic microorganisms, yeast and molds, and Enterobacteriaceae. With the combination of heating and starvation, microbes were no longer detectable.

Table 3 shows that the aw-value of different breeding substrates were within a very small range, so it can be assumed that there is too little freely available water for rapid growth of microorganisms. The pH value, also shown in Table 3, was between 5 and 7.2. This pH range allows the growth of many microorganisms. Molds, yeasts, and lactic acid bacteria in particular can grow in a slightly acidic environment (pH 5–6). As a result, the analyzed microbiological load is related to the microorganisms on the mealworms themselves as well as to the microbial load of their feces.

In conclusion, larvae of yellow mealworm grown on different substrates showed a microbial load between 7.41 ± 1.18 cfu × g^−1^ and 9.47 ± 2.05 cfu × g^−1^. The composition of the substrate showed no effect on the growth of microorganisms in the mealworms grown on it. To reduce the number of microorganisms on fresh mealworms, heating in combination with previous defecation seems to be the preferable method.

## 4. Discussion

Due to the discussion about the use of insects as an alternative dietary source of protein, this study makes a valuable contribution in evaluating the safety of mealworm larvae as food for human nutrition. In humans, the impact of diet on the gut microbiota is a widely studied phenomenon [18]; however, in insects there have only been a few investigations in recent years. Studies on insects’ microbiome can improve the production settings for insect rearing and can also influence safe consumption. Thus, an accurate and reliable identification of the microbiota of edible insects is needed in order to evaluate the possible presence of pathogens or spoilage agents as well as beneficial microbes [7]. As Cesaro et al. and Garofalo et al. stated, microorganisms in edible insects represent one of the major concerns for public health and can contaminate the external cuticle of an insect as well as its gut through the ingestion of contaminated feed [4,19]. This fact highlights the importance of studies investigating the insects as well as their diet and frass.

Montalbán et al. [20] showed that the gut microbiota profile of *Tenebrio molitor* was affected by diet. Mealworms were fed with three formulations corresponding to three different levels of starch and protein. *Spiroplasma* spp. and *Lactobacillus* spp. were associated with a higher starch content, and the abundance of *Staphylococcus* spp. and *Leuconostoc* spp. was associated with a higher protein content in the diet of *T. molitor*.

Osimani et al. analyzed fresh mealworm larvae, their feeding substrate (wheatmeal), and frass [8]. In this case, low microbial contamination of the wheatmeal was detected, whereas larvae and frass were characterized by high loads of Enterobacteriaceae, lactic acid bacteria, and several species of mesophilic aerobes; more precisely, *Enterobacter* spp., *Erwinia* spp., *Enterococcus* spp., and *Lactococcus* spp. were found as dominant species in the larvae and frass, whereas *Klebsiella* spp., *Pantoea* spp., and *Xenorhabdus* spp. Were found to be in the minority. Spore-forming bacteria were detected to a lesser extent in all the samples. Although pathogens like *Listeria* spp. and *Salmonella* spp. were not detected, the authors discourage the consumption of fresh mealworm larvae and recommend the examination of the most opportune processing methods (boiling, frying, drying).

Microbiological contamination of five different feeding substrates (brewery spent grains, bread and cookie leftovers, and mixes of brewer’s spent grain or bread with cookies) and larvae’s microbial loads (starved and unstarved larvae) were determined by Mancini et al. [13]. It was found that microbial loads were partially affected by the fed diet. In the unstarved larvae, staphylococci, yeasts and molds, and bacterial endospores were significantly different depending on the diet, whereas in starved larvae, the diets only affected yeasts and molds and bacterial endospores. The substrates were also analyzed, but the microbiological amounts were lower than the detection limits. Microbiological analyses highlighted the total absence of *Escherichia coli* and *Bacillus cereus* as well as the absence of *Listeria monocytogenes* and *Salmonella* spp. [13].

Garofalo et al. analyzed whole, dried mealworm larvae and found a great bacterial diversity, but relatively low counts of total mesophilic aerobes, Enterobacteriaceae, lactic acid bacteria, *Clostridium perfringens* spores, yeasts, and molds. Furthermore, several gut-associated bacteria were identified, some of which may act as opportunistic pathogens in humans, including food spoilage bacteria, as well as *Spiroplasma* spp. Viable pathogens such as *Salmonella* spp. were not detected but the presence of *Listeria* spp., *Staphylococcus* spp., *Clostridium* spp., and *Bacillus* spp. (in low abundance) was discovered [7].

Megido et al. studied whole, fresh *T. molitor* larvae from European farms and evaluated the efficiency of different processing methods (blanching, freeze-drying, sterilization) in reducing microorganism counts. According to the authors, all untreated samples had high total mesophilic aerobic bacteria, as well as yeast and mold counts. Nevertheless, the processing treatments led to a reduction of the total counts and attained a reduction of microorganisms under the required limits. These results confirmed that fresh larvae need a processing step before consumption [10].

Vandeweyer et al. analyzed microbial counts of fresh mealworm larvae from different rearing companies and different production batches. They found that the microbial counts from the fresh insects were generally high. Even different rearing batches from a single rearing company showed differences in microbial counts, especially for aerobic bacterial endospores, aerobic psychrotrophic organisms, and yeasts and molds, with the counts varying markedly. *Salmonella* spp. and *Listeria* spp. were not detected in any of the samples. All in all, no overall differences between rearers could be observed [11].

The aim of the study of Wynants et al. was to investigate whether transmission of *Salmonella* spp. to mealworms can occur in the event that mealworms are fed with contaminated wheat bran as the substrate. When larvae were present, however, the survival of *Salmonella* spp. in larvae and bran depended on the contamination level; when present at a low level in the substrate, *Salmonella* spp. were not retained by the larvae during the seven-day period, likely either because of competitive exclusion by the endogenous larval microbiota and/or because of the antibacterial activity of the larvae [3].

Stoops et al. characterized the microflora (total viable aerobic count, Enterobacteriaceae, lactic acid bacteria, yeasts and molds, and bacterial endospores) of fresh edible mealworm larvae and found high microbial counts and a variety of potential spoilage bacteria and food pathogens. In contrast to the study of Vandeweyer et al., different insect batches resulted in quite similar microbial numbers (except for bacterial endospores). The results of this study suggest that a subsequent processing step is required to avoid or minimize risks involved with the consumption of edible insects [11,14].

In summary, these studies show the presence of Enterobacteriaceae as well as varying levels of microbial load in mealworm larvae. No viable pathogens such as *Salmonella* spp. could be detected. Klunder et al. also highlighted that the major risk for safety issues derives from the ingestion of the gastrointestinal tract of insects [9]. A further processing step is recommended by the majority of the authors to reduce the microbial load of larvae of *Tenebrio molitor.* As Wynants et al. and Mancini et al. showed, starvation resulted in the reduction of artificially contaminated (with *Salmonella* spp. And *Listeria monocytogenes*) substrates [3,21]. In an additional study by Mancini et al., a starvation treatment for 24 h affected the bacterial endospore content without influencing other microorganisms [22].

## 5. Conclusions

The results of this study, in the context of most scientific literature, showed that the choice of substrate has no influence on the microbial composition of the mealworm larvae. In general, mealworm larvae are known for their stable and species-specific microbiota, and have been shown to be frequently associated with Enterobacteriaceae, such as *Enterobacter* spp., *Klebsiella* spp., *Erwinia* spp., and *Pantoea* spp.; lactic acid bacteria, namely *Enterococcus* spp., and *Lactococcus* spp.; as well as *Staphylococcus* spp., *Bacillus* spp., *Pseudomonas* spp., and *Clostridium* spp. In contrast to other edible insects’ species, *Lactobacillus* spp., *Streptococcus* spp., *Pediococcus* spp., *Weissella* spp., *Salmonella* spp., *Cronobacter* spp., *Haemophilus* spp., *Listeria* spp., *Vibrio* spp., *Yersinia* spp., *Morganella* spp., *Rickettsiella* spp., and *Propionibacterium* spp. are less frequently observed [4].

However, Montalbán et al. [20] found different genera of microorganisms in larvae fed with varying diets, and in a very recent paper, Khanal et al. [23], showed significant differences in bacterial communities between fed and fasted groups of mealworm larvae.

The focus of this work was to investigate as many different by-product streams as possible and their potential suitability as substrates for growing insects using the example of the yellow mealworm as an alternative protein source. The microbial analysis was therefore limited to the mesophilic aerobic total plate count, and the presence of molds, yeasts, and enterobacteria. The main focus was on the analysis of a possible correlation between the different by-product streams, their microbial load, and the microbial load of the mealworms grown on them. This work clearly showed that the investigated by-product streams are well suited as substrates and that there is no correlation between the individual parameters investigated. Furthermore, the second focus was on the evaluation of different processing steps to reduce the general microbiological load of mealworm larvae.

Nevertheless, the congruent findings further demand the need for more detailed investigations into *Tenebrio molitor* as a safe food choice. Moreover, to date, there are no specific microbiological criteria for insect products sold for human consumption.

Most data clearly demonstrate that essential processing steps are required to reduce the number of microorganisms [4,9,10,11,14,24]. In conclusion, these procedures allow mealworm larvae to be used as safe alternative sources of protein in food.

In contrast to these findings, further research is needed to explore the relationship between rearing conditions, including hygiene, and the insect microbiota, as also mentioned by Vandeweyer et al. [11]. Another research objective in this context could be the analysis of mealworm excretions. The frass of *T. molitor* is dry and scentless, and thus is less likely to become a source of pollution and pathogens compared with the excreta of many other animals. The microbial load of the frass, on one hand, as well as the organic and inorganic components on the other, would thus provide a complete picture of the use of the yellow mealworm as an adequate and safe food source for human nutrition.

## Figures and Tables

**Table 1:** Microbial analysis of larvae of *Tenebrio molitor* and ten breeding substrates (mean value and standard deviation in log cfu × g^−1^).

Substrate	TAC *T. molitor*	EB *T. molitor*	YM *T. molitor*	TAC Substrate	EB Substrate	YM Substrate
N	8.26 ± 1.86	6.85 ± 1.12	n.d.	8.46 ± 1.12	6.96 ± 1.03	n.d.
B	7.41 ± 1.18	6.85 ± 0.99	n.d.	7.63 ± 1.29	6.37 ± 1.17	n.d.
D	9.06 ± 1.59	8.43 ± 1.61	n.d.	8.79 ± 1.88	7.73 ± 1.28	n.d.
H	8.57 ± 1.31	6.39 ± 0.96	n.d.	6.89 ± 0.86	5.95 ± 0.76	n.d.
OMCG	8.83 ± 1.31	8.41 ± 2.04	n.d.	8.34 ± 1.59	6.85 ± 1.01	6.25 ± 0.79
WB	9.47 ± 2.05	8.09 ± 1.26	6.14 ± 0.64	7.68 ± 1.33	5.44 ± 0.88	6.47 ± 0.95
CM	7.89 ± 1.16	6.42 ± 0.98	n.d.	7.52 ± 1.61	7.17 ± 0.86	n.d.
CGM	9.39 ± 1.03	7.37 ± 1.20	n.d.	6.53 ± 1.04	6.13 ± 1.10	n.d.
MRP	8.83 ± 1.49	7.46 ± 1.16	n.d.	8.21 ± 1.12	7.46 ± 0.98	n.d.
PSOC	9.06 ± 1.35	8.31 ± 1.18	n.d.	7.48 ± 1.28	6.90 ± 1.01	n.d.

Abbr.: (N) nettle, (B) bread remains, (D) draff, (H) hemp seed oil cake, (OMCG) oyster mushrooms with coffee grounds, (WB) wheat bran, (CM) chestnut breakage and meal, (CGM) corn germ meal, (MRP) malt residual pellets, (PSOC) pumpkin seed oil cake, (TAC) total mesophilic aerobic bacterial counts, (EB) Enterobacteriaceae, (YM) yeasts and molds.

**Table 2:** Microbial evaluation of processing methods of larvae of *Tenebrio molitor* (mean value and standard deviation in log cfu × g^−1^).

Processing Method	TAC	EB	YM
D/H	0 ± 0	0 ± 0	0 ± 0
D/NH	7.69 ± 1.45	5.64 ± 0.41	5.25 ± 0.43
ND/H	4.19 ± 1.04	3.80 ± 1.04	0 ± 0
ND/NH	8.93 ± 1.07	6.28 ± 0.56	5.41 ± 0.83

Abbr.: (D) defecated for 24 h, (H) heated, (ND) non-defecated, (NH) non-heated, (TAC) total mesophilic aerobic bacterial counts, (EB) Enterobacteriaceae, (YM) yeasts and molds.

**Table 3:** Water activity (aw-value) and pH of breeding substrates for growth of *Tenebrio molitor*.

Substrate	aw-Value	pH
N	0.208	7.2
B	0.1967	5.0
D	n.d.	n.d.
H	0.2499	6.3
OMCG	0.2091	5.5
WB	0.2122	5.9
CM	0.2250	5.4
CGM	0.2286	6.2
MRP	0.2603	5.9
PSOC	0.2063	6.0

Abbr.: (N) nettle, (B) bread remains, (D) draff, (H) hemp seed oil cake, (OMCG) oyster mushrooms with coffee grounds, (WB) wheat bran, (CM) chestnuts breakage and meal, (CGM) corn germ meal, (MRP) malt residual pellets, (PSOC) pumpkin seed oil cake; n.d. not determined.

## Data Availability

The data presented in this study are available on request from the corresponding author.
